# Multi-environment prediction of suicidal beliefs

**DOI:** 10.3389/fpsyt.2024.1425416

**Published:** 2024-11-18

**Authors:** Austin V. Goddard, Audrey Y. Su, Yu Xiang, Craig J. Bryan

**Affiliations:** ^1^ Department of Electrical and Computer Engineering, The University of Utah, Salt Lake City, UT, United States; ^2^ Department of Biostatistics, Brown University, Providence, RI, United States; ^3^ Department of Psychiatry and Behavioral Health, The Ohio State University, Columbus, OH, United States

**Keywords:** suicide, domain adaptation, causal inference, invariance, primary care

## Abstract

Suicide disproportionately affects the military and veteran population, yet the task of identifying those at an increased risk of suicidal behavior remains challenging. In the face of this complex issue, novel machine learning methods have been applied to study the relationship between suicide and potential risk factors, but are often not generalizable to new and unseen samples. Herein, we examine the problem of prediction on unknown environments, commonly known as environment-wise domain adaptation, as it relates to the prediction of suicidal beliefs, measured with items from the Suicide Cognitions Scale (SCS). We adapt several recently invariance-based models trained using a sample consisting of people without any prior suicidal ideation (SI) to the prediction of suicidal beliefs of those with prior SI. In addition, we examine the possible causal relations regarding the SCS. Using a prospective sample of 2744 primary care patients with 17 risk and protective factors, we show that, to some extent, these methods are able to generalize to a new environment, namely, a sample with prior SI. Additionally, our results indicate suicidal ideation and suicidal behavior are likely to be causal children of SCS.

## Introduction

From 1999-2022, the suicide rate in the United States increased by more than 33%, rising from 10.5 per 100,000 to 14.3 per 100,000 (1). Suicide remains one of the leading causes of death in the United States, especially among military personnel and veterans (2). As a result, identifying those who are at an increased risk of suicidal behavior (SB) is an important task. Even though many factors of suicidal behavior have been identified, the complex relationship between suicidal behavior and its many factors can make prediction difficult (3, 4). This is partly because the direct causes of suicide remain unknown. Researchers have further posited that efforts to identify suicide’s causes are hampered by the construct’s complexity and heterogeneity. Suicide can, for example, occur in the presence of mental illness but also in its absence (5–7), as a reaction to a life stressor or in the context of no obvious precipitant (8, 9), and be impulsive or carefully planned (10–14).

Owing to these complexities, researchers have argued that suicide is best understood as a complex adaptive system composed of multiple interacting and interdependent components, such that suicide is greater than the sum of its parts (3). Consistent with this perspective, machine learning has been increasingly used to analyze the complex relationship among suicide and its many factors (15, 16). While machine learning has proven useful for modeling nonlinear and interactive relationships among multiple risk and protective factors, machine learning models rarely replicate across samples, limiting their clinical utility in general practice settings (17–19). While controlled experiments like randomized clinical trials can offer insights into the causes of suicidal behavior, this approach is often expensive and infeasible to employ with sufficient speed and scale, implicating the potential value of alternative approaches. Novel computational methods that can reliably identify those at risk of suicidal behavior across diverse samples are therefore needed.

Accumulating research shows that the assessment of the *suicidal belief system*, typically measured with items from the Suicide Cognitions Scale (SCS), is a clinically useful method for identifying patients who will attempt suicide (20, 21). According to Rudd (22), the suicidal belief system is a network of severely negative beliefs and perceptions about the self, others, and the world that confers psychological vulnerability to experiencing suicidal states. Multiple studies show that SCS items distinguish patients who will attempt suicide from those who will not, even when accounting for suicidal ideation (15, 23–25). Newer studies further show that SCS items also distinguish patients who will attempt suicide from those who will experience suicidal ideation only, (20) suggesting the suicidal belief system may causally relate to suicidal *behavior* as an outcome distinct from suicidal *thinking*.

We wish to leverage this observation as a basis for applying several recently proposed methods to predict the suicidal belief system in unseen environments. Such a prediction task is often referred to as environment-wise domain adaptation (26–28). Like machine learning methods, these methods analyze complicated relationships among many predictor variables and an outcome. Moreover, they are able to generalize across many domains, allowing robust predictions in new environments. Specifically, we test the usefulness of such approaches by selecting the test environment (those who reported prior suicidal ideation) and the training environment (those who did not report prior suicidal ideation). The goal is to extend a model built on the training environment to predict the test environment reliably. Successful prediction of the suicidal belief system in the test environment will highlight the advantages of multi-environment domain adaption methods for identifying suicidal behaviors and further solidify it as an important factor (and possible causal predictor) of suicidal behavior and suicidal ideation. To achieve this goal, we examined a unique class of prediction methods aimed toward identifying individuals who are at an increased risk of suicidal behavior in new environments. The data used in this work was collected as part of the PRImary care Screening Methods (PRISM) study, a longitudinal cohort observation study aimed at testing various methods for identifying primary care patients who would later attempt suicide (24).

## Methods

### Participants and procedures

Participants were 2744 primary care patients ranging from 18 to 89 (M=40.4, SD=19.6) years of age. Patient characteristics are summarized in **Table 1**. Patients were eligible to participate if they were at least 18 years old, eligible to receive medical services from the Department of Defense, able to understand and read the English language, and able to complete the informed consent process. Patients were excluded if they had a medical or psychiatric condition that diminished their ability to provide informed consent (e.g., acute intoxication, psychosis). Participants were recruited and enrolled from the waiting rooms of six primary care and family medicine clinics from July 2015 to August 2018. Because we did not have approval to maintain information about patients who declined to participate, the overall refusal rate is unknown. Study procedures were approved by the Naval Health Research Center Institutional Review Board (NHRC.2014.0046).

**Table 1:** Sample demographics.

Variable	n (%)
Gender	
Male	1380 (51.3)
Female	1279 (47.5)
Other	9 (0.3)
Prefer not to Answer	17 (0.6)
Unknown/Missing	59 (2.2)
Race	
White/Caucasian	1811 (67.3)
Black/African American	506 (18.8)
Asian	115 (4.3)
Native Amer./Alaska Native	123 (4.6)
Pac. Isl./Native Hawaiian	44 (1.6)
Other	272 (10.1)
Hispanic/Latino Ethnicity	
Yes	415 (15.4)
No	2199 (81.7)
Other	20 (0.7)
Prefer not to Answer	51 (1.9)
Prior Suicidal Ideation	774 (28.8)
Prior Suicidal Behavior	238 (8.8)
Prior Nonsuicidal Self-Injury	312 (11.6)

### Predictor variables

Seventeen empirically supported risk and protective factors were selected as candidate causal variables. The instruments used to assess these variables are described below with internal consistency estimates (i.e., Cronbach’s alpha) derived from the present sample. Amongst these variables, the target variable SCS is also described.

**Guilt, shame, and inward hostility.** The Differential Emotions Scale-IV (DES-IV; 29) is a 36-item self-report scale that assesses 12 distinct emotional states with 3 items each. Items are rated using a 5-point scale and summed, with higher scores indicating greater frequency of experiencing each emotion. In this study, items measuring guilt, shame, and inward hostility were measured. Cronbach’s alphas in the current sample were 0.92 for guilt, 0.89 for shame, and 0.92 for inward hostility. The guilt, shame, and inward hostility items can be completed in less than 2 minutes.

**Internal and external entrapment.** The Entrapment Scale (ES; 30) is a 16-item self-report scale that assesses external (10 items; e.g., “I am in a situation I feel trapped in”) and internal (6 items; e.g., “I want to get away from myself”) entrapment. Items are rated using a 5-point scale and summed, with higher scores indicating more severe levels of external and internal entrapment. Cronbach’s alphas in the current sample were 0.95 for external entrapment and 0.94 for internal entrapment. The ES can typically be completed in less than 3 minutes.

**Social support.** The short form of the Interpersonal Support Evaluation List (ISEL-12; 31) is a 12-item self-report scale that assesses three facets of social support (4 items each): the perceived availability of advice or guidance from others (appraisal support), feelings of empathy and acceptance from others (belonging), and material help or assistance (tangible support). Items are rated using a 5-point scale and summed, with higher scores indicating stronger perceptions of each type of support. Cronbach’s alphas in the current sample were 0.68 for appraisal support, 0.72 for belonging, and 0.57 and tangible support. The ISEL-12 can typically be completed in less than 2 minutes.

**Positive and negative affect.** The International Positive and Negative Affect Scale-Short Form (I-PANAS-SF; 32) is a 10-item self-report scale that assesses positive affect (5 items) and negative affect (5 items). Items are rated using a 5-point scale and summed, with higher scores indicating more intense positive or negative experience. Cronbach’s alphas in the current sample were 0.86 for positive affect and 0.88 for negative affect. The I-PANAS-SF can typically be completed in less than 1 minute.

**Posttraumatic stress symptoms.** The Primary Care Posttraumatic Stress Disorder Checklist (PC-PTSD; 33) is a 4-item self-report scale designed to screen for a diagnosis of PTSD using items that assess the presence or absence of PTSD symptoms within the past month. Items are rated using a yes/no response format and summed, with more yes responses indicating increased probability of a PTSD diagnosis. The Kudar-Richardson estimate in the current sample was 0.86. The PC-PTSD can typically be completed in less than 1 minute.

**Depression symptoms.** The Patient Health Questionnaire Depression Subscale (PHQ-8; 34) is a 9-item self-report scale that assesses the frequency of the symptoms of a major depressive episode during the past two weeks. Items are rated using a 4-point scale and summed, with higher scores indicating greater frequency of each symptom. Item 9, which assesses frequency of suicidal ideation within the past two weeks, was omitted from the calculation of the total scale score so we could examine suicidal ideation as an independent predictor of suicidal behavior. Cronbach’s alpha for the full scale in the current sample was 0.90. The PHQ-9 can typically be completed in less than 2 minutes.

**Suicidal ideation.** The ninth item of the PHQ-9 assesses the frequency of “thoughts that you would be better off dead, or thoughts of hurting yourself in some way” within the past two weeks. The item is rated using a 4-point scale, with higher scores indicating more frequent suicidal ideation. This item can typically be answered in less than 10 seconds.

**Fearlessness about death.** The Acquired Capability for Suicide Scale-Fearlessness About Death (ACSS-FAD; 35) is an 8-item self-report scale that assesses fear of death (e.g., “The fact that I am going to die does not affect me”). Items are rated using a 5-point scale and summed, with higher scores indicating less fear of death (i.e., more fearlessness). Cronbach’s alpha in the current sample was 0.71. The ACSS-FAD can typically be completed in less than 2 minutes.

**Reasons for living.** The Brief Reasons for Living Inventory (BRFLI; 36) is a 14-item self-report scale that assesses adaptive beliefs and expectations for living. Items are rated using a 6-point rating scale and summed, with higher scores indicating a stronger motive to not attempt suicide. Cronbach’s alpha in the current sample was 0.89. The BRFLI can typically be completed in less than 3 minutes.

**Sleep disturbance.** The Insomnia Severity Index (ISI; 37) is a 7-item self-report scale that assesses the severity of sleep disturbance and the impact of sleep disruption on one’s life within the past week. Items are rated using a 5-point scale and summed, with higher scores indicating greater subjective sleep disturbance. Cronbach’s alpha in the current sample was 0.93. The ISI can typically be completed in less than 1 minute.

**Suicidal belief system.** The Suicide Cognitions Scale-Revised (SCS-R; 38) is a 16-item self-report scale designed to measure maladaptive suicidogenic beliefs and perceptions commonly reported by suicidal patients (e.g., “I can’t stand this pain anymore” and “Nothing can help solve my problems”). Items are rated using a 5-point scale and summed, with higher scores indicating greater propensity to attempt suicide. Cronbach’s alpha in the current sample was 0.97. The SCS-R can typically be completed in less than 3 minutes.

### Variable selection

It is common for multi-environment domain adaptation methods to have a high complexity when the number of predictors is large. Thus, it is infeasible to use the 17 possible predictors available from the PRISM study (24). Instead, lasso variable selection was used to select the subset of variables summarized in **Table 2**, where we adopted the conventional ten-fold cross-validation to choose the regularization parameter in lasso.

**Table 2:** Variables selected by lasso variable selection.

Variable
I-PANAS-SF negative affect
PHQ-9 suicide ideation item
PHQ-9
ISEL appraisal
ES external
ES internal
DES-IV inward hostility

### Environment selection

As mentioned, the test and training environments are chosen to be prior suicidal ideation (prior SI) and no prior SI, respectively. We selected prior SI for our environments because it is commonly used in clinical settings as an indicator of elevated risk for suicidal behavior. Multi-environment domain adaptation methods also require the training data to be partitioned into distinct environments. We partitioned according to the demographic variables GENDER, AGE, and RACE (White vs. non-White) because suicidal behaviors are known to vary in frequency across these subgroups. The variable Age was used to evenly split the training environment in two using a threshold of 28 years. We considered prior suicide attempts (versus no prior attempts) as alternative test and train environments but there were an insufficient number of prior attempt cases after we partitioned by demographics to run our analyses.

### Data analytic approach

The methods used to predict in unknown environments rely on detecting models that are invariant across environments. Given an index set ε of environments, the general assumption is that the conditional distribution of the target variable $Y^{e}$ given its direct causes $X^{e}$ are invariant with respect to each environment e∈ε . This assumption follows closely with ideas concerning causality and is often referred to as ‘autonomy’ or ‘modularity’ (39–41) as well as ‘stability’ (41, 42). Thus, simply put, when we say some variable $X^{e}$ is a parent of a child variable $Y^{e}$ , we mean the relationship $Y^{e}|X^{e}$ is invariant over all possible environments e∈ϵ (regarding what constitutes a possible environment, see (43).

Each method used requires the relationship between the target and other variables to be linear. This becomes an issue as the two possible children we considered, SB and SI, were binary variables. To overcome this issue, we propose to vary each method slightly by using the log odds transformation on these variables. The log-odds (logarithm of the odds) of a logistic regression model with response Z and predictors X is defined as


$$log\left(\frac{p\left(x\right)}{1-p\left(x\right)}\right)=\beta_{0}+X\mathrm{\beta ,}$$

where p(x)=P(Z=1|X=x) , β is a vector of coefficients, and $\beta_{0}$ is the intercept term. Most importantly to us, the log odds are linear in the predictors X . Thus, such a transform from binary variables into continuous ones allows us to apply a wide band of methods that require a linear relationship between the target and other variables, which is the main assumption we need in our approach. A summary of the log odds transformation procedure is outlined in - **Algorithm 1.** We employ this procedure in both the testing and the training environments before any prediction method is used. For the training environment, the predictors used were those in **Table 2** as well as SCS-R. In the testing environment, the target SCS was not available. Thus, the log odds were estimated using only the predictors in **Table 2**. To the best of our knowledge, this type of transform on binary variables has not been explored in a multi-environment regression setting.

Algorithm 1 **Log odds transform.**

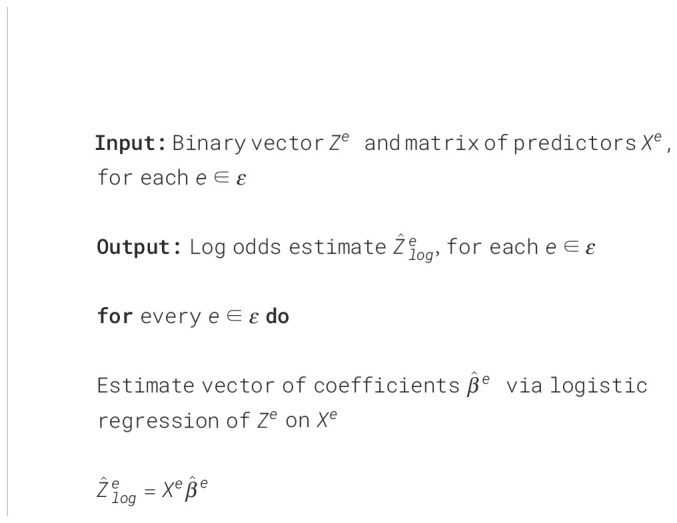

We now discuss the various methodologies used to estimate the target (SCS-R) in unseen environments in greater detail. Each method relies on estimating various models given different subsets of suicidal predictors. For each case, we use ordinary least squares (OLS) as the model estimate.

**Invariant Matching Property (IMP).** When the invariance principle mentioned above is violated, a series of recent work called IMP (44–47) takes advantage of invariances relating to child nodes as well as parent nodes of the target. These methods use a novel invariant relationship, referred to as the IMP, involving a child of the target to identify invariant models. This approach to detect invariant models is unique in that it is able to detect invariances amidst interventions on the target variable. Models are accepted based on an invariance score determined by how closely the models follow the IMP. Additionally, models are rejected based on a prediction score, meaning only the best invariant models are used. Invariant models are identified using a possible child of the target and a subset of all possible predictors. All accepted models are then used to predict on unknown environments.

**IMP_inv_.** The IMP_inv_ (45) method can be considered a variant of the IMP method, as they share the same basic structure and assumptions. The two methods vary in how invariance is scored. Specifically, the IMP_inv_ method is scored by testing that the IMP model’s residual distribution is invariant over environments.

In addition to the predictor variables mentioned (**Table 2**), the IMP and IMP_inv_ methods also require variables to act as child nodes. In this setting, two possible choices of the child variable are considered. These include whether or not participants reported suicidal behavior (SB) or suicidal ideation (SI) over the duration of the study.

**Invariant Causal Prediction (ICP).** Previously referred to as Method II (48), the ICP method relies solely on invariance between parent and target and identifies invariant models by testing that the residual distribution of each linear model is invariant over environments. Each invariant model corresponds to an accepted set of predictors. Prediction in unknown environments occurs only using the model given by the intersection of all accepted sets. While this allows ICP to control the family-wise error rate of falsely selecting at least one correct causal parent, it also makes the method conservative, often returning no invariant predictors (an empty intersection).

**Stable Regression (SR).** Like IMP and IMP_inv_, SR (28) takes a less conservative approach to invariant prediction compared to ICP. SR introduces a *stable blanket*, which is the set of predictors containing all information about the target $Y^{e}$ . The stable blanket relies on a weaker form of invariance dependent on expectation where $E[Y^{e}|X^{e}]$ = $E[Y^{h}|X^{h}]$ for all e,h ∈ ε . While SR identifies invariant models similarly to ICP based on the residual distribution, it also selects models that are not only invariant but also have high prediction power.

## Results

In addition to IMP, IMP_inv_, ICP, and SR, we also compare with ordinary least squared (OLS). While an OLS-trained model will not extend well to unknown environments, it serves as a good baseline in examining whether other methods are able to detect proper invariances. The MATLAB platform and coding language are used to run all experiments. To ensure there is no missing data, a participant is removed from this analysis if data does not exist for each risk and protective factor.

For the various methods, we present the mean squared error (MSE) of each SCS-R estimator using test environment (with prior SI) and training environments (no prior SI). We present results for settings when the child node is SI (see **Table 3**) and SB (see **Table 4**). For almost all training environments, IMP, IMP_inv_, and SR report a smaller MSE compared to that of OLS. This implies, at least to a certain degree, that these methods were able to generalize to the test environment. The one case where IMP_inv_ performed worse than OLS is for the environment Age. ICP was unable to produce any invariant predictors in this setting, in line with the conservative nature of this method (21). When the child node was SI, the SR method was able to generalize to some degree in all environments except Age. Particularly, when the child node was SB, the SR method outperformed IMP and IMP_inv_.

**Table 3:** Mean Squared Error results on the test environment when the child is SI.

Training Environments	IMP	IMP_inv_	SR	ICP	OLS
AGE	**51.9**	73.6	61.1	NA	59.5
GENDER	60.8	**51.9**	52.2	NA	62.7
RACE	58.2	54.6	**52.2**	NA	63.9

We report NA if no invariance was identified. Bold values indicate the highest Mean Squared Error over all methods.

**Table 4:** Mean Squared Error results on the test environment when the child is SB.

Training Environments	IMP	IMP_inv_	SR	ICP	OLS
AGE	55.6	52.1	**52.0**	NA	58.1
GENDER	54.6	52.2	**52.1**	NA	56.3
RACE	52.8	59.7	**52.2**	NA	56.5

We report NA if no invariance was identified. Bold values indicate the highest Mean Squared Error over all methods.

As mentioned in Section 2, the IMP and IMP_inv_ approaches require access to a causal child of the target variable in order to predict in unknown environments. We observed that in all cases, the MSE of either IMP or IMP_inv_ is lower than that of OLS. This further strengthens the belief that SCS-R is an important factor and possible causal predictor of SB and SI. IMP can also be used in predicting causal parents using a voting procedure (49). In this approach, variables with more votes are more likely to be causal predictors. Results of causal parent discovery using IMP can be seen in **Tables 5**–**8**. While there is no clear parent of SCS-R, the variable ISEL appraisal consistently received the highest number of votes over environments and can be considered the most likely causal predictor of SCS-R.

**Table 5:** Votes for possible parents of SCS-R using IMP and SI as the child node.

Variable	AGE	GENDER	RACE
I-PANAS-SF negative affect	1	3	33
PHQ-9 suicide ideation item	1	3	9
PHQ-9	1	3	41
ISEL appraisal	1	0	53
ES external	1	2	25
ES internal	1	3	36
DES-IV inward hostility	1	2	12

**Table 6:** Votes for possible parents of SCS-R using IMP_inv_ and SI as the child node.

Variable	AGE	GENDER	RACE
I-PANAS-SF negative affect	1	3	35
PHQ-9 suicide ideation item	1	3	9
PHQ-9	1	3	43
ISEL appraisal	1	0	55
ES external	1	2	18
ES internal	1	3	38
DES-IV inward hostility	1	2	13

**Table 7:** Votes for possible parents of SCS-R using IMP and SB as the child node.

Variable	AGE	GENDER	RACE
I-PANAS-SF negative affect	5	93	3
PHQ-9 suicide ideation item	5	51	1
PHQ-9	5	48	3
ISEL appraisal	5	135	3
ES external	1	57	3
ES internal	3	44	3
DES-IV inward hostility	3	53	3

**Table 8:** Votes for possible parents of SCS-R using IMP_inv_ and SB as the child node.

Variable	AGE	GENDER	RACE
I-PANAS-SF negative affect	9	78	2
PHQ-9 suicide ideation item	9	43	1
PHQ-9	9	38	2
ISEL appraisal	8	115	2
ES external	3	37	2
ES internal	4	40	2
DES-IV inward hostility	3	46	2

## Discussion

Suicide has remained a leading cause of death among veterans and military personnel for many years, but the complex factors contributing to suicide remain poorly understood, due in part to the limitations of existing predictive modeling techniques like OLS and machine learning that are not generalizable to new samples and environments. In light of these challenges, novel computational methods that overcome non-generalizability have great potential to advance our understanding of suicide. In this study, we compared multiple domain adaptation techniques (IMP, IMP_inv_, SR, and ICP) to OLS for the prediction of the suicidal beliefs system: a variable that reliably differentiates patients who will attempt suicide from those who will not, indicating it is closely related to the processes and mechanisms underlying suicide. Our results show that the IMP, IMP_inv_, and SR methods generally performed better than OLS and achieved smaller mean squared errors. This suggests that, at least to some extent, these domain adaptation techniques can better generalize a suicide-prediction model developed in one environment to new, unknown environments.

Our findings derived from the IMP and IMP_inv_ methods also support a possible causal role of suicidal beliefs, measured in this study with the SCS-R, as especially important factors related to, and possible causal predictors of, suicidal behavior. This conclusion is based on the IMP and IMP_inv_ methods’ requirement for an available child node to accurately predict unknown environments. In this study, suicidal ideation and suicidal behavior were examined as possible child nodes. When these two factors were included as child nodes, SCS-R scores could be predicted in unknown environments. This implies that suicidal beliefs are probably causally related to suicidal ideation and suicidal behavior. Additional research is needed to further test this possibility, but generally align with previous research showing that mental health treatments that directly target these beliefs can reduce suicide risk. Brief cognitive behavioral therapy (BCBT) for suicide prevention, for instance, is a psychotherapy that has been shown to significantly reduce suicide attempts (50, 51). In BCBT, clinicians teach patients how to identify their suicidal beliefs and use cognitive reappraisal skills to reduce the intensity of these beliefs and replace them with more balanced and less extreme beliefs.

Our results also implicate the potential value of the IMP heuristic voting procedure for identifying potential causal predictors of variables. Although no single predictor or set of predictors received an overwhelming majority of votes in this study, the appraisal subscale of the ISEL consistently received the most votes across training environments, suggesting it was more likely than all other variables to be causally related to suicidal beliefs. Because appraisal support involves the perceived availability of people who provide advice and guidance (52), our findings suggest that deficits in this particular dimension of social support may be an especially important contributor to heightened vulnerability for suicidal behavior.

Given the importance of appraisal support found in this study, we recommend that military clinical strategies regarding mental health should promote a greater emphasis on this factor. In comparison to civilian populations, male veterans tend to have less diverse social networks and report significantly lower appraisal social support ISEL scores than their male civilian counterparts, while female veterans have smaller social networks than female civilians (53). Furthermore, high levels of baseline PTSD in military populations have been linked to low levels of appraisal support (54). On the other hand, a significant, negative correlation has been found between received social support and disorders such as major depressive disorder, generalized anxiety disorder, PTSD, and suicidal ideation in US military populations (55). Altogether, our findings, combined with that of existing literature, suggest that the formal integration of a strong social support network into the treatment process may be effective in improving the mental health of VA patients. Appraisal/social support has already been well-established as an important facilitator of good mental health in various contexts, such as substance recovery (in which many current protocols involve inviting a friend or a relative to attend treatment sessions), thought to be due to the protective, stress-buffering effect of social support in the face of distressing events (56–59). Thus, for the VA, outreach efforts that improve perceived appraisal support, such as the Compassionate Contact Corps in which socially isolated veterans speak to volunteers, should be prioritized.

Nevertheless, conclusions based on the present study should be made cautiously considering several study limitations. First, our study was conducted in U.S. military medical clinics with adult patients. Replication of these results in non-military settings is therefore needed before definitive conclusions for the general population can be made. Likewise, our results may not generalize to pediatric or youth samples. Our study also relied on self-report methodology, which can be vulnerable to motivated and socially desirable responding. Future research using a variety of complementary assessment methods would be valuable for further testing the utility of domain adaptation techniques.

Despite these limitations, our results provide useful information about the potential utility of domain adaptation techniques, a novel computational approach that could overcome the limitations of widely used but non-generalizable machine learning methods including logistic regression. Additional work to further develop and refine domain adaptation techniques is therefore warranted.

## Data Availability

The raw data supporting the conclusions of this article will be made available by the authors upon reasonable request. Requests to access the datasets should be directed to CB.
